# Objective and Self-Reported Factors Associated With Food-Environment Perceptions and Fruit-And-Vegetable Consumption: A Multilevel Analysis

**DOI:** 10.5888/pcd11.130324

**Published:** 2014-03-27

**Authors:** Sean C. Lucan, Amy Hillier, Clyde B. Schechter, Karen Glanz

**Affiliations:** Author Affiliations: Amy Hillier, School of Design, University of Pennsylvania, Philadelphia, Pennsylvania; Clyde B. Schechter, Department of Family and Social Medicine, Albert Einstein College of Medicine, Bronx, New York; Karen Glanz, Perelman School of Medicine and School of Nursing, University of Pennsylvania, Philadelphia, Pennsylvania.

## Abstract

**Introduction:**

Few studies have assessed how people’s perceptions of their neighborhood environment compare with objective measures or how self-reported and objective neighborhood measures relate to consumption of fruits and vegetables.

**Methods:**

A telephone survey of 4,399 residents of Philadelphia, Pennsylvania, provided data on individuals, their households, their neighborhoods (self-defined), their food-environment perceptions, and their fruit-and-vegetable consumption. Other data on neighborhoods (census tracts) or “extended neighborhoods” (census tracts plus 1-quarter–mile buffers) came from the US Census Bureau, the Philadelphia Police Department, the Southeastern Pennsylvania Transportation Authority, and the federal Supplemental Nutrition Assistance Program. Mixed-effects multilevel logistic regression models examined associations between food-environment perceptions, fruit-and-vegetable consumption, and individual, household, and neighborhood characteristics.

**Results:**

Perceptions of neighborhood food environments (supermarket accessibility, produce availability, and grocery quality) were strongly associated with each other but not consistently or significantly associated with objective neighborhood measures or self-reported fruit-and-vegetable consumption. We found racial and educational disparities in fruit-and-vegetable consumption, even after adjusting for food-environment perceptions and individual, household, and neighborhood characteristics. Having a supermarket in the extended neighborhood was associated with better perceived supermarket access (adjusted odds ratio for having a conventional supermarket, 2.04 [95% CI, 1.68–2.46]; adjusted odds ratio for having a limited-assortment supermarket, 1.28 [95% CI, 1.02–1.59]) but not increased fruit-and-vegetable consumption. Models showed some counterintuitive associations with neighborhood crime and public transportation.

**Conclusion:**

We found limited association between objective and self-reported neighborhood measures. Sociodemographic differences in individual fruit-and-vegetable consumption were evident regardless of neighborhood environment. Adding supermarkets to urban neighborhoods might improve residents’ perceptions of supermarket accessibility but might not increase their fruit-and-vegetable consumption.

## Introduction

Several studies have considered associations between neighborhood food environments and dietary intake, particularly consumption of fruits and vegetables (1). Studies have used various methods to measure food environments, including objective measures (eg, measured distance to food stores [2,3]) and self-reported measures (eg, perceptions of food-store accessibility [4,5]). Few studies have used both objective and self-reported measures (6–9), however, even though both types of measures may be important to fruit-and-vegetable consumption. Self-reported measures may provide the clearest picture of people’s perceptions of their environment; objective measures may provide concrete targets for environmental interventions to promote greater fruit-and-vegetable intake.

Beyond distinctions between self-reported and objective measures of food environments, it may also be important to consider broader individual and environmental characteristics that could contribute to perceptions of fruit-and-vegetable accessibility and to levels of purchasing and consumption. For instance, having a high density of food stores that sell fruits and vegetables may matter little if transportation is not adequate to access those stores, if neighborhood crime makes shopping at those stores unsafe, or if the produce available in those stores does not meet individual preferences.

Several studies on fruit-and-vegetable intake have examined individual and neighborhood characteristics simultaneously using multilevel models (5–13). Few of these studies, however, have considered both objective and self-reported measures of food environments (6–9). Those that have generally have not given attention to some potentially relevant individual characteristics (eg, overall health status, body mass index [BMI]), and none have considered broader neighborhood issues (eg, public transportation, neighborhood crime).

Our study builds on prior research, considering and adjusting for several relevant individual, household, and neighborhood factors, to assess 1) how individuals’ self-reported perceptions compare with objective measures of neighborhood environments, and 2) how self-reported and objective neighborhood measures each relate to reported fruit-and-vegetable consumption.

## Methods

The University of Pennsylvania institutional review board approved this study.

### Survey data on individuals

Data on individuals came from Public Health Management Corporation’s biennial random-digit–dialed Southeastern Pennsylvania Household Health (SPHH) survey (www.chdbdata.org/householdsurvey.html). The 2010 survey, administered from June through October, included 4,399 adult respondents from Philadelphia’s 379 census tracts. Service-area stratification helped ensure sufficient representation of sociodemographic subpopulations. Response rates were 25.5% for landlines and 20.6% for cellular telephones, comparable to rates from similar large community health surveys (14).

### Dependent variables in multilevel models

Depending on the regression model, the dependent variable was either individuals’ reported fruit-and-vegetable consumption or one of 3 perceptions of the neighborhood food environment. Fruit-and-vegetable consumption was measured by a single item: “How many servings of fruits and vegetables do you eat on a typical day? A serving of a fruit or vegetable is equal to a medium apple, half a cup of peas or half a large banana.” Response options were open-ended (any nonnegative integer). Perceptions of the neighborhood food environment related to 1) supermarket accessibility (“Do you HAVE to travel outside of your neighborhood to go to a supermarket?” [yes, no]), 2) grocery quality (“How would you rate the overall quality of groceries available in the stores in your neighborhood?” [excellent, good, fair, poor, absent]), and 3) produce availability (“How easy or difficult is it for you to find fruits and vegetables in your neighborhood?” [very easy, easy, difficult, very difficult]). 

### Self-reported independent variables and covariates

The dependent variables also served as predictors in models for which they were not the outcomes. Studies show associations among these variables (4,6,12,15,16) and between these variables and modeling covariates. Modeling covariates included characteristics of individuals (age, sex, race/ethnicity, nativity [whether or not born in the United States], education, health status, BMI [calculated from self-reported height and weight]) (5,6,9–13,15,16), their households (number of other adults at home, whether children lived at home, annual income, poverty level, food insecurity in previous 12 months, use of public assistance) (6,16), and their neighborhoods (an index of social capital) (17), all derived from the SPHH survey. Poverty level was provided by the survey vendor and was determined from an imputed income variable based on the employment status of the main wage earner and the respondent’s education level. The question on food insecurity asked, “In the past 12 months, did you or other adults in your household ever cut the size of your meals or skip meals because there was not enough money in the budget for food?” The index of social capital was provided by the survey vendor and determined by answers to questions about 1) the number of neighborhood groups or organizations, 2) the likelihood of neighbors helping each other, 3) a personal feeling of being part of the neighborhood, 4) agreeing neighbors can be trusted, and 5) whether neighbors ever work together; on an additive 10-point scale — with each of the 5 component questions having responses ranging in value from 0 to 2 — low was 1–4, medium was 5–7, high was 8–10. 

Other neighborhood covariates included the percentage of racial/ethnic minority populations (18,19) from the 2010 US Census (www.socialexplorer.com/data/C2010) and the 2006–2010 American Community Survey (ACS) (www.socialexplorer.com/data/ACS2010_5yr/documentation/c4387efd-2717-444d-a4eb-20d7ed25ba14) and education, poverty, and vehicle ownership (18–24) from the 2006–2010 ACS. For census and ACS data, “neighborhoods” were defined by census tracts: small, relatively permanent, statistical subdivisions of counties commonly used to measure neighborhoods in food-environment research (1). For SPHH data, the term “neighborhood” in survey questions was open to respondent interpretation.

### Objectively measured independent variables

Objective independent variables included neighborhood crime rates, availability of public transportation, and supermarket presence. Neighborhood crime data (total drug and violent crime arrests by neighborhood per 10,000 residents) came from the Philadelphia Police Department (www.opendataphilly.org/opendata/resource/215/philadelphia-police-part-one-crime-incidents/). Public transportation data (subway or trolley stops in the neighborhood [because bus access had too little variability to be distinguishing]) came from the Southeastern Pennsylvania Transportation Authority (www.pasda.psu.edu/uci/SearchResults.aspx?originator=Southeastern PennsylvaniaTransportationAuthority&Keyword=&searchType=originator&entry=PASDA&sessionID=6921113201383013230). Data on supermarket presence came from the federal Supplemental Nutrition Assistance Program retailer database (http://snap-load-balancer-244858692.us-east-1.elb.amazonaws.com/ArcGIS/rest/services/retailer/MapServer). Supermarkets were of one of 2 types: limited-assortment (smaller, restricted selection) or conventional. Supermarket presence and public transportation were assessed in neighborhoods (census tracts) and “extended neighborhoods” (the 1-quarter mile extending beyond census-tract boundaries in all directions).

### Statistical analyses

We used Stata version 12.1 (StataCorp LP, College Station, Texas) to calculate descriptive statistics and answer our study questions. Only 3 variables were missing more than 2.5% of data: reported fruit-and-vegetable consumption (4.2%), the index of social capital (18.7%), and household income (23.3%). We used multiple imputation by chained equations (25) to impute the former 2 variables before running analytic models. Household income was not imputed (and not included in models) because missing values were not likely to be missing at random; other socioeconomic variables (ie, education, household poverty, food insecurity, and public assistance) were included. We used mixed-effects multilevel regression models to assess how self-reported perceptions of neighborhoods compared with objective measures of neighborhoods. Models regressed each of one of the 3 food-environment perceptions (supermarket accessibility, produce availability, and grocery quality) on both of the 2 remaining perceptions and on all of the objective neighborhood measures (crime rates, public transportation, and supermarket presence), considering and adjusting for individual, household, and neighborhood covariates. Similar models assessed how environmental measures related to reported fruit-and-vegetable consumption, regressing fruit-and-vegetable consumption on all other study variables. All models used logistic regression (ordered logistic regression when dependent variables were polychotomous) to generate odds ratios (ORs), applying the appropriate survey weights to account for the SPHH survey’s complex sampling and graphically confirming proportional odds assumptions in all cases. We modeled ordinal predictors as continuous variables because bivariable plots with outcomes generally suggested linear relationships with the variables as coded. 

## Results

For the survey sample of 4,399 respondents poststratified to the 2010 US Census, the median age was 45 years, and 54.6% were women (Table 1). The population included almost as many non-Hispanic whites (39.5%) as non-Hispanic blacks (41.0%); 86.3% were born in the United States. About half (49.3%) had no schooling beyond high school and rated their health as less than very good (51.7%). A third (33.0%) were overweight; nearly another third (30.9%) were obese. About a quarter (23.8%) did not live with other adults; two-thirds (66.0%) lived without children. Almost half (48.8%) had annual household incomes of $50,000 or less. Nearly a quarter (22.2%) lived below the federal poverty level, 15.2% were food insecure, and more than one-third (36.3%) received public assistance. More than one-quarter (26.5%) reported living in neighborhoods with low levels of social capital. A great majority (89.8%) reported consuming fewer than 5 servings of fruits and vegetables per day. For food-environment perceptions, 29.5% reported a need to travel outside their neighborhood to get to a supermarket, 8.3% reported produce was hard or very hard to find in their neighborhood, and 23.6% reported that grocery quality was less than good.

**Table 1:** Self-Reported Consumption of Fruits and Vegetables, Perceptions of Neighborhood^a^ Food Environment, and Covariates From the 2010 Southeastern Pennsylvania Household Health (SPHH) Survey

Self-reported variable from survey	Weighted % (n = 4,399)^b^
**Covariates in All Regression Models**	
**Individual characteristics**	
**Age, y**	
<30	23.0
30–44	26.6
45–59	27.7
≥60	22.6
**Sex**	
Female	54.6
Male	45.4
**Race/ethnicity**	
Non-Hispanic white	39.5
Non-Hispanic black	41.0
Hispanic/Latino	10.9
Other^c^	6.5
**Foreign born**	
No	86.3
Yes	13.2
**Education**	
≥College graduate	29.6
Some college	20.5
High school graduate	36.5
<High school graduate	12.8
**Health status**	
Excellent	19.9
Very good	28.3
Good	29.9
Fair or poor	21.8
**Body mass index (kg/m^2^)**	
<25.0	33.4
25.0–29.9 (overweight)	33.0
≥30.0 (obese)	30.9
**Household characteristics**	
**No. of other adults at living at home**	
0	23.8
1	46.6
≥2	29.3
**Children living at home**	
No	66.0
Yes	33.9
**Annual household income,^d^ $**	
≤25,000	30.5
25,001–50,000	18.3
50,001–100,000	20.0
>100,000	8.8
**Poverty level**	
>200% Federal poverty level	58.1
100%–200% Federal poverty level	19.6
<100% Federal poverty level	22.2
**Food insecurity in past 12 months**	
No cutting meal size or meal	84.4
Yes, cutting meal size or meal	15.2
**Any public assistance^e^**	
No	60.6
Yes	36.3
**Neighborhood characteristics**	
**Index of social capital^f^**	
Low	26.5
Medium	44.4
High	12.0
**Alternating as Dependent Variable (Depending on Regression Model)**	
**Fruit-and-vegetable consumption**	
**Fruit or vegetable servings eaten on a typical day** g	
0–1	27.3
2	28.6
3	20.1
≥4	19.9
**Perceptions of neighborhood** a ** food environment**	
**Supermarket accessibility in neighborhood** a	
Have a supermarket in neighborhood^a^	70.1
Must travel outside of neighborhood^a^ to get to a supermarket	29.5
**Produce availability in neighborhood** a	
Very easy to find	55.7
Easy to find	34.7
Hard or very hard to find	8.3
**Grocery quality in neighborhood** a	
Excellent	31.3
Good	43.7
Fair, poor, or absent	23.6

a “Neighborhood” not defined in SPHH survey.

b SPHH survey variables were poststratified to 2010 US Census values for Philadelphia by age category, sex, and racial/ethnic groups; participants are representative of all of Philadelphia (n = 1,172,744). Percentages for categorical variables may not sum to 100% because of missing data. All variables had between 0% and 2.5% missing data, except for reported fruit-and-vegetable consumption (4.2% missing), household income (23.3% missing), and the index of social capital (18.7% missing).

c For race/ethnicity, “other” was a heterogeneous category including Asian, multiracial, and Native American.

d Data on household income included in table to describe sample but not included in regression models (see footnote a, Table 3).

e Types of public assistance included Supplemental Nutrition Assistance Program (SNAP), otherwise known as the federal Food Stamps program; Special Supplemental Nutrition Program for Women, Infants, and Children (WIC); Social Security’s Supplemental Security Income (SSI); Social Security Disability Insurance (SSDI); and Temporary Assistance for Needy Families (TANF).

f Index of social capital in SPHH survey included questions about 1) number of neighborhood groups or organizations, 2) likelihood of neighbors helping each other, 3), a personal feeling of being part of the neighborhood, 4) agreeing neighbors can be trusted, and 5) whether neighbors ever work together. On a 10-point scale, low, 1–4 points; medium, 5–7 points; high, 8–10 points.

g Based on the SPHH survey, 5.6% of Philadelphians consumed 0 servings of fruits or vegetables on a typical day, and 10.2% typically consumed ≥5 servings.

Demographics (including vehicle ownership) varied by neighborhood, as did crime rates (Table 2). Nearly 21% of neighborhoods had a subway or trolley stop (compared with 40.1% of extended neighborhoods). Conventional supermarkets were available in about one-third fewer neighborhoods than limited-assortment supermarkets (13.7% vs 21.6%), and in about one-quarter fewer extended neighborhoods (46.4% vs 63.1%).

**Table 2:** Neighborhood^a^ Covariates and Objectively Measured Independent Variables From Various Sources, Philadelphia, 2010

Variable	Values for All Philadelphia Neighborhoods (n = 379)
**Neighborhood Covariates**	
**Demographics from US Census, 2010**	
Racial/ethnic minorities, mean % (1st–99th percentile range)	62.1 (5.5–99.6)
**Demographics from American Community Survey, 2006–2010**	
Hispanic, mean % (1st–99th percentile range)	10.9 (1.1–81.7)
Foreign born, mean % (1st–99th percentile range)	10.8 (0–38.2)
Did not graduate from high school, mean % (1st–99th percentile range)	20.5 (0.5–51.8)
<100% of Federal poverty guidelines, mean % (1st–99th percentile range)	25.3 (2.2–69.1)
Households with no vehicle, mean % (1st–99th percentile range)	34.4 (2.6–71.7)
**Objectively Measured Independent Variables**	
**Crime rates from Philadelphia Police Department, 2010**	
No. of drug and violent crime arrests in neighborhood per 10,000 residents, mean (1st–99th percentile range)	7.1 (0–233)
**Public transportation data from Southeastern Pennsylvania Transportation Authority, 2010**	
Subway or trolley stop in neighborhood, % (subway or trolley stop in extended neighborhood^b^, %)	20.8 (40.1)
**Data on supermarket presence from Supplemental Nutrition Assistance Program, 2010**	
Larger conventional supermarket in neighborhood, % (larger conventional supermarket in extended neighborhood^b^, %)	13.7 (46.4)
Any supermarket^c^ in neighborhood, % (any supermarket^c^ in extended neighborhood^b^, %)	21.6 (63.1)

a “Neighborhood” defined as US Census tract.

b “Extended neighborhood” defined as census tract plus an extending 1-quarter–mile buffer in all directions.

c “Any supermarket” defined as both larger conventional supermarket and smaller limited-assortment store.

Table 3 shows results from multilevel models (in which, importantly, variable categorizations differ in some cases from those shown in Table 1). Perceived supermarket accessibility was directly related to perceived produce availability (OR, 1.76), perceived grocery quality (OR, 1.97), and having either a conventional (OR, 2.04) or limited-assortment supermarket (OR, 1.28) in the extended neighborhood; inverse associations were found with age (OR, 0.99), food insecurity (OR, 0.55), public assistance (OR, 0.74), and mean percentage of households in the neighborhood having no vehicle (OR, 0.99). Perceived produce availability was directly related to perceived supermarket accessibility (OR, 1.90), perceived grocery quality (OR, 2.35), health status (OR, 1.18), social capital (OR, 1.25), and rates of neighborhood crime (OR, 1.07); inverse associations were found with household poverty (OR, 0.85), food insecurity (OR, 0.71), and neighborhood poverty (OR, 0.98). Perceived grocery quality was directly associated with perceived supermarket accessibility (OR, 2.41), perceived produce availability (OR, 2.54), age (OR, 1.02), health status (OR, 1.16), BMI (OR, 1.11), and social capital (OR, 1.27); inverse associations included “other” race (OR, 0.57) and the mean percentage of racial/ethnic minorities in the neighborhood (OR, 0.99). Direct associations with reported fruit-and-vegetable consumption included female sex (OR, 1.73), level of education (OR, 1.30), health status (OR, 1.19), and neighborhood social capital (OR, 1.36); inverse associations were found with non-Hispanic black race/ethnicity (OR, 0.65), household poverty (OR, 0.87), and having a subway or trolley stop in the extended neighborhood (OR, 0.80).

**Table 3:** Multilevel Regression Models Examining Associations of Food-Environment Perceptions and Reported Fruit-And-Vegetable Consumption, Philadelphia, 2010^a^

Variables and Covariates^b^	Model 1: Dependent Variable, Supermarket Accessibility		Model 2: Dependent Variable, Produce Availability		Model 3: Dependent Variable, Grocery Quality		Model 4: Dependent Variable, Fruit-and-Vegetable Consumption	
	OR (95% CI)	*P*	OR (95% CI)	*P*	OR (95% CI)	*P*	OR (95% CI)	*P*
**Southeastern Pennsylvania Household Health Survey**								
**Fruit-and-vegetable consumption**								
Progressively greater no. of servings on typical day	0.94 (0.88–1.02)	—	1.06 (0.99 1.14)	—	0.99 (0.93–1.05)	—	NA	
**Food-environment perceptions**								
Supermarket accessibility (supermarket in neighborhood vs must travel to get to supermarket)	NA		1.90 (1.57–2.31)	<.001	2.41 (2.00–2.90)	<.001	0.87 (0.73–1.03)	—
Produce availability (progressively easier to find)	1.76 (1.51–2.05)	<.001	NA		2.54 (2.20–2.93)	<.001	1.11 (0.98–1.27)	—
Grocery quality (progressively better quality)	1.97 (1.71–2.28)	<.001	2.35 (2.06–2.70)	<.001	NA		0.97 (0.86–1.09)	—
**Individual covariates**								
Age (increasing 1-year increments)	0.99 (0.98–0.99)	<.001	1.00 (0.99–1.01)	—	1.02 (1.01–1.02)	<.001	1.00 (1.00–1.01)	—
Female sex	0.93 (0.76–1.12)	—	0.85 (0.72–1.02)	—	1.02 (0.87–1.19)	—	1.73 (1.49–2.01)	<.001
Race/ethnicity (vs non-Hispanic white)								
Non-Hispanic black	1.10 (0.82–1.48)	—	1.00 (0.77–1.31)	—	0.85 (0.67–1.09)	—	0.65 (0.51–0.82)	<.001
Hispanic (all races)	0.82 (0.54–1.25)	—	0.98 (0.66–1.46)	—	0.94 (0.64–1.36)	—	0.84 (0.59–1.20)	—
Other	0.76 (0.49–1.18)	—	0.94 (0.61–1.44)	—	0.57 (0.38–0.83)	.004	0.76 (0.54–1.07)	—
Education (increasing schooling)	0.91 (0.83–0.99)	.04	1.01 (0.93–1.10)	—	1.00 (0.92–1.08)	—	1.30 (1.21–1.40)	<.001
Health status (progressively better)	0.96 (0.87–1.05)	—	1.18 (1.09–1.29)	<.001	1.16 (1.07–1.26)	.001	1.19 (1.11–1.29)	<.001
BMI (increasing BMI)	1.05 (0.93–1.18)	—	1.00 (0.90–1.12)	—	1.11 (1.01–1.23)	.04	1.07 (0.97–1.17)	—
**Household covariates**								
Poverty (progressively greater)	0.98 (0.85–1.14)		0.85 (0.75–0.97)	.02	0.88 (0.77–1.00)	—	0.87 (0.78–0.98)	.02
Food insecurity in past 12 months (yes vs no)	0.55 (0.42–0.72)	<.001	0.71 (0.55–0.91)	.008	0.83 (0.65–1.06)	—	0.86 (0.69–1.09)	—
Any public assistance (yes vs no)	0.74 (0.59–0.93)	.009	0.98 (0.80–1.19)	—	1.19 (0.98–1.44)	—	0.96 (0.82–1.14)	—
**Neighborhood covariate**								
Social capital (progressively better)	1.13 (0.95–1.33)	—	1.25 (1.07–1.45)	.005	1.27 (1.10–1.47)	.001	1.36 (1.19–1.55)	<.001
**Neighborhood Covariates from US Census and American Community Survey**								
Racial/ethnic minorities, mean %	1.00 (0.98–1.00)	—	1.00 (0.99–1.00)	—	0.99 (0.99–1.00)	.001	1.00 (1.00–1.01)	—
<100% Federal poverty level, mean %	1.01 (1.00–1.02)	—	0.98 (0.97–1.00)	.009	0.99 (0.98–1.00)	—	1.00 (0.99–1.01)	—
Households with no vehicle, mean %	0.99 (0.98–0.99)	.002	1.00 (0.98–1.01)	—	1.00 (1.00–1.01)	—	1.00 (1.00–1.01)	—
**Objective Independent Neighborhood Variables From Various Sources**								
Rate of drug and violent crimes^c^	0.96 (0.91–1.01)	—	1.07 (1.01–1.12)	.01	0.97 (0.92–1.02)	—	1.02 (0.97–1.07)	
SEPTA stop in extended neighborhood^d^^, ^ e	0.91 (0.74–1.13)	—	1.16 (0.95–1.41)	—	0.88 (0.73–1.07)	—	0.80 (0.68–0.96)	.01
Conventional supermarket in extended neighborhood^d^^, ^ f	2.04 (1.68–2.46)	<.001	1.01 (0.84–1.20)	—	0.95 (0.80–1.11)	—	0.96 (0.82–1.11)	—
Limited-assortment market in extended neighborhood^d^^, ^ f	1.28 (1.02–1.59)	.03	1.04 (0.85–1.27)	—	0.92 (0.76–1.11)	—	1.00 (0.84–1.20)	—

Abbreviations: OR, odds ratio: CI, confidence interval; —, *P* value ≥ .05; NA, not applicable (not included in model); BMI, body mass index; SEPTA, Southeastern Pennsylvania Transit Authority.

a ORs are from multilevel regression models using multiple imputation estimates. Household income was not imputed (and not included in models) because missing values were not likely to be missing at random (ie, people at either income extreme may have been less likely to report their income); other socioeconomic variables (ie, education, household poverty, food insecurity, and public assistance) were included. Logistic regression was used for dichotomous outcomes, ordered logistic regression for polychotomous outcomes. For parsimony, not shown are the ORs for being foreign-born, having other adults at home, having children at home, mean percentages of Hispanic residents, foreign-born residents, and residents with less than a high school education; these variables all had nonsignificant ORs that were near unity (ie, 1.00) in all models.

b Variables categorized as in Table 1 for all modeling except the following: fruit-and-vegetable consumption (modeled as 5 categories: 0, 1, 2, 3, ≥4), produce availability (modeled as 4 categories: very easy, easy, hard, very hard to find), grocery quality (modeled as 5 categories: excellent, good, fair, poor, absent), education (modeled as 5 categories: < high school graduate, high school graduate, some college, college graduate, postcollege), health status (modeled as 5 categories: excellent, very good, good, fair, poor).

c Source: Philadelphia Police Department, 2010.

d “Extended neighborhood” defined as census tract plus an extending 1-quarter–mile buffer in all directions.

e Source: SEPTA, 2010.

f Source: Supplemental Nutrition Assistance (or Food Stamps) Program, 2010.

## Discussion

In our analysis of food environments, objective and self-reported measures were not entirely concordant with each other; for example, an objective measure of having a supermarket in the extended neighborhood was associated with better perceived supermarket accessibility but not produce availability or grocery quality. No neighborhood perceptions were related to fruit-and-vegetable consumption. There were nonintuitive associations between some variables in models. Sociodemographic differences in fruit-and-vegetable consumption were evident even after adjusting for individual, household, and neighborhood factors.

Research shows that perceptions of neighborhood food environments are strongly and directly related to each other (4) but may not be strongly or directly related to more objective measures of neighborhood environments. For instance, whereas Moore et al showed a direct association between the perceived availability of healthful foods and store density (26), Caspi et al showed a 31.5% mismatch between measured distance to the nearest supermarket and perceived accessibility (6), Williams et al showed poor correlation between several perceived and objective aspects of neighborhood food environments (ie, accessibility, variety, and cost) (27), and Gustafson et al showed an inverse correlation between the perceived availability of healthful foods and the number of stores selling them (8). The critical point may be that all food stores are not equal. Our analyses showed that supermarket presence (objectively measured) was related to perceived accessibility but not produce availability or grocery quality, suggesting that even if supermarkets are present in a neighborhood, they may not satisfy individual preferences or prompt individuals to shop there.

Positive perceptions of food environments may relate to greater self-efficacy for consuming fruits and vegetables (28). Whether such perceptions relate to reported fruit-and-vegetable consumption is unclear, but earlier multilevel modeling by a member of our team suggested no strong relationships (5). Other multilevel modeling, in different settings using different measures, showed that only a few tested perceptions (of affordability, selection, and accessibility) were directly associated with produce consumption, each with varying degrees of significance for fruits versus vegetables (10,12). A study considering fruits and vegetables together showed perceptions of produce selection and quality — but not affordability — were directly associated with fruit-and-vegetable consumption (13), whereas another study found that none of these perceptions was associated with consumption (29). All of these studies were limited by not considering objective measures of the food environment.

Objective measures of the food environment, along with self-reported measures, were considered in a few studies using multilevel models (6–9). Caspi et al showed that objective distance to a supermarket was not associated with fruit-and-vegetable intake (6), consistent with our findings. Although the Caspi study did show that perceiving a supermarket within walking distance of home was associated with greater reported fruit-and-vegetable intake, the effect size was small (0.5 servings/day) (6). Caldwell et al showed that greater perceived and objective availability were both associated with increased fruit-and-vegetable consumption (7). Although our perception measure was the same as in the Caldwell study, we considered the neighborhood (supermarket presence) as opposed to the store (produce display space) for our objective measure, and we adjusted for multiple factors excluded from the Caldwell models (models that included only sex, age, and dietary intake) (7). Gustafson et al showed that strongly agreeing that a neighborhood has many healthful foods was not associated with fruit and vegetable intake and that living in neighborhoods with a supercenter and convenience store was associated with lower intake (8). These findings suggest that store type matters, consistent with differences we found related to conventional versus limited-assortment supermarkets. Zenk et al showed that neither satisfaction with neighborhood produce nor measured distance to the nearest supermarket was associated with fruit-and-vegetable consumption (9). The Zenk findings are most consistent with our own; however, the Zenk study also showed that when only large grocery stores were considered, having such a store in the neighborhood was associated with consuming 0.69 more daily servings of fruits and vegetables (9). Our analyses did not show a difference in fruit-and-vegetable consumption by store type but did show that larger (conventional) supermarkets were associated with better perceptions of supermarket accessibility than smaller (limited-assortment) supermarkets.

A study by Ball et al considered only objective measures of the food environment and showed that store density did not relate to fruit-and vegetable consumption, nor did it mediate relationships with socioeconomic considerations (11). Our analyses likewise showed no relationship between objective measures of stores and fruit-and-vegetables consumption but did show racial and socioeconomic differences. These differences were not explained by different perceptions of food environments, as others have suggested (12); racial and educational disparities in fruit-and-vegetable consumption were evident in our analyses, even after adjusting for food-environment perceptions and several potentially confounding individual, household, and environmental factors.

Our analyses also found differences in neighborhood food-environment perceptions by sociodemographic characteristics. We found better perceptions of some aspects of neighborhood food environments with younger age; better reported health; not being poor, receiving public assistance, or being food insecure; and living in neighborhoods with greater social capital and less poverty. These issues may relate to people’s mobility, social support, material resources, and ability to travel and shop for food, all of which may constrain conceptualizations of “neighborhood.” For instance, wealthier, healthier people who own or have access to cars might have a more expansive concept of neighborhood such that having a supermarket in the “extended neighborhood” was directly associated with perceptions of produce accessibility, whereas having a supermarket in the census tract only was not. We found better perceptions of some aspects of neighborhood food environments among people who had a greater BMI, who were not a member of a racial/ethnic minority group, and who lived in neighborhoods with fewer minorities. These issues may relate to personal, social, and cultural ideas about eating. For example, even food that is available and high in quality might not be perceived well if the offerings do not satisfy cultural preferences.

Our study produced 2 counterintuitive findings. One was that perceptions of produce availability increased with increasing rates of neighborhood crime. The other was that having a subway or trolley stop in the neighborhood was associated with lower fruit-and-vegetable consumption. Both of these findings could have been due to chance. The former, being of small magnitude and marginal significance, is difficult to rationalize. The latter, however, might be explained by the fact that people may not use public transportation for food shopping (30). Neighborhoods with subway and trolley stops in our analyses had lower numbers of residents who owned a personal vehicle; thus subway and trolley stops might be a marker for lower rates of vehicle ownership and less ability to get to stores selling produce and high-quality groceries.

Our study had several strengths. First, it used population-based individual data from a large urban area and integrated neighborhood data from diverse sources. Second, analyses used rigorous statistical methods with appropriate survey weights and multiple imputation for missing data. Third, analyses modeled several individual, household, and neighborhood characteristics, including both self-reported and objective measures not considered in prior research. Additionally, the analyses examined 2 definitions of neighborhood and 2 definitions of supermarket. Finally, dependent variables included both food-environment perceptions and reported fruit-and-vegetable consumption to assess how both related to different individual, household, and neighborhood characteristics.

Despite methodological strengths, our analyses had limitations. First, the design was cross-sectional, precluding assessment of causality or directionality for the associations found. Second, because of multiple tested associations, nominal *P* values may not reflect the actual potential for false positive error. Third, the survey variables of interest were all derived from single-item questions, but, reassuringly, studies that used more detailed food-frequency questionnaires and screeners to assess dietary intake reported similar results (8,9,29), and a study testing similar measures of food-environment perceptions showed high reliability, particularly in urban areas (31). Although response rates for the SPHH survey used in our study were far from perfect, evidence increasingly challenges the presumed correlation between response rate and survey bias (14). Another limitation was that survey questions did not assess additional aspects of food environments, such as affordability or cost (10,12,15,16) or the competing accessibility of less healthful foods (15,16). Finally, geographic boundaries used to define neighborhoods may not have aligned precisely with survey respondents’ conceptualizations of their neighborhoods’ borders.

Our analyses found poor correspondence between objective and self-reported measures of neighborhoods and produced some nonintuitive findings on food environments and fruit-and-vegetable consumption. Key associations that emerged depended on definitions of neighborhoods and supermarkets and also on individual sociodemographic characteristics, suggesting that strategies to improve neighborhood food environments and individuals’ diets will require nuance. Although our analyses were cross-sectional, findings do not support certain proposed food-environment modifications to increase produce consumption. Adding supermarkets — even larger stores with wider selections — to urban neighborhoods might improve residents’ perceptions of supermarket accessibility but might not increase their fruit-and-vegetable consumption.
